# Mean Platelet Volume Is Related to Cumulative Disease Damage in Patients with Systemic Lupus Erythematosus

**DOI:** 10.3390/life14040428

**Published:** 2024-03-22

**Authors:** Yolanda Fernández-Cladera, Marta Hernández-Díaz, María García-González, Juan C. Quevedo-Abeledo, Adrián Quevedo-Rodríguez, Fuensanta Gómez-Bernal, Cristina Gómez-Moreno, Candelaria Martín-González, Miguel Á. González-Gay, Iván Ferraz-Amaro

**Affiliations:** 1 Division of Central Laboratory, Hospital Universitario de Canarias, 38320 Tenerife, Spain; yolanda.fernandezcladera@gmail.com (Y.F.-C.); fuensanta95@gmail.com (F.G.-B.); 2 Division of Rheumatology, Hospital Universitario de Canarias, 38320 Tenerife, Spainmargagon23@hotmail.com (M.G.-G.); 3 Division of Rheumatology, Hospital Doctor Negrín, 35010 Las Palmas de Gran Canarias, Spain; quevedojcarlos@yahoo.es (J.C.Q.-A.); adrian-ce@hotmail.es (A.Q.-R.); 4 Fundación Jiménez Díaz, School of Nursing of Madrid, Autonomous University of Madrid, 28040 Madrid, Spain; cgomezm@fjd.es; 5 Department of Internal Medicine, Universidad de La Laguna (ULL), 38200 Tenerife, Spain; mmartgon@ull.edu.es; 6 Division of Rheumatology, Instituto de Investigación Sanitaria, Fundación Jiménez Díaz, 28040 Madrid, Spain; 7 Department of Medicine and Psychiatry, University of Cantabria, 39005 Santander, Spain

**Keywords:** mean platelet volume, systemic lupus erythematosus

## Abstract

Mean platelet volume (MPV), which represents the average platelet size in femtoliters, has emerged as a reliable biomarker in several systemic and chronic disorders. However, its relationship with disease characteristics in large series of patients with systemic lupus erythematosus (SLE) has not been exhaustively studied to date. In the present work, we aimed to analyze how disease characteristics, including disease activity and cumulative damage, relate to MPV in a well-characterized series of SLE patients. In total, 179 patients with SLE and 181 age- and sex-matched healthy controls were recruited. Complete blood counts including MPV were assessed. Linear multivariable analysis was performed to evaluate the relationship between MPV and SLE disease characteristics, including composite scores of disease activity and damage. MPV was significantly lower in patients with SLE compared to controls after multivariable analysis (beta coefficient, −0.7 [95% confidence interval, −1.1 to −0.3)] fL, *p* < 0.001). Although the SLEDAI disease activity index was not related to MPV, the SLICC score measuring cumulative disease damage was significantly associated with lower MPV values after adjustment for covariates. Elements of the SLICC score that were associated with lower MPV levels were those pertaining to the kidney, peripheral vascular, and musculoskeletal manifestations of the disease. In conclusion, MPV is lower in patients with SLE compared to matched controls. This MPV downregulation is primarily due to the renal, peripheral vascular and musculoskeletal manifestations of the disease. MPV may represent a biomarker of accrual disease damage in SLE.

## 1. Introduction

The mean platelet volume (MPV) represents the average platelet size, measured in femtoliters (fL). This metric is calculated based on the volume of platelets circulating in the bloodstream and exhibits an inverse relationship with the platelet count. This is because the body maintains control over the overall mass of platelets, prioritizing this regulation over individual platelet numbers or sizes [1]. An elevated or decreased MPV can sometimes be indicative of certain hematological medical conditions or disorders. For example, a high MPV indicates active platelet production in the bone marrow, as in immune thrombocytopenia. Elevation of MPV is also seen in some inherited disorders of platelet function and in some myelodysplastic syndromes. On the contrary, a diminished MPV serves as an indicator of bone marrow suppression, as observed in conditions such as aplastic anemia [2]. Additionally, MPV has gained interest as a biomarker in other systemic inflammatory or chronic disorders [3]. For example, disrupted MPV values have been linked to mental disorders [4], cardiovascular disease [5] and cancer [6].

Systemic lupus erythematosus (SLE) is a persistent autoimmune disorder of unknown origin that has the potential to affect almost any organ in the body. Immune dysregulation, particularly the generation of multiple antinuclear antibodies, is a prominent feature of the condition [7]. People with SLE exhibit various clinical symptoms, ranging from mild joint and skin problems to more serious complications involving the kidneys, blood, or central nervous system. Moreover, hematologic irregularities are frequently encountered in SLE, both at the point of diagnosis and throughout the disease’s progression. Principal hematologic manifestations in SLE encompass anemia, leukopenia, thrombocytopenia, lymphadenopathy, and/or splenomegaly [8]. These abnormalities may manifest as a direct result of SLE, be associated with another concurrent medical condition, or be induced by a treatment used for SLE.

To our knowledge, there are no previous reports that have studied how MPV relates to disease manifestations, including disease activity and damage, in large series of SLE patients. For this reason, in the present work, we aimed to analyze how the characteristics of the disease are related to MPV in a well-characterized cohort of patients with SLE.

## 2. Materials and Methods

### 2.1. Study Participants

A cross-sectional study enrolled 360 participants, consisting of 179 individuals diagnosed with systemic lupus erythematosus (SLE) and 181 age- and gender-matched healthy controls. The inclusion criteria for patients with SLE required being 18 years of age or older, having a clinical diagnosis of SLE, and meeting the classification criteria established by the 2019 American College of Rheumatology (ACR). In this sense, although most of them had been diagnosed before 2019, they underwent retrospective testing to meet the 2019 classification criteria [9]. These patients, diagnosed by rheumatologists, received regular follow-ups at rheumatology outpatient clinics. Participants on prednisone, at an equivalent dose of ≤10 mg/day, were eligible, considering the common use of glucocorticoids in SLE treatment. Healthy controls, sourced from the community, were recruited by general practitioners in primary care settings. Individuals with a history of any inflammatory rheumatic disease were excluded from the control group, and none of the controls were undergoing glucocorticoid treatment. Neither patients nor controls had a history of hematological diseases such as aplasia or myeloproliferative disorders, and they were not receiving iron supplementation or had acute thrombosis. The research adhered to the principles of the Declaration of Helsinki. The study protocol obtained approval from the Institutional Review Committee of Hospital Universitario de Canarias and Hospital Universitario Doctor Negrín (both in Spain), and written informed consent was obtained from all participants (Approval Number 2020-123).

### 2.2. Data Collection

Participants in the study filled out a questionnaire regarding cardiovascular risk factors and medication usage, in addition to undergoing a thorough physical examination. Measurements of weight, height, body mass index, abdominal circumference, and systolic and diastolic blood pressure (recorded with the participant in the supine position) were conducted under standardized conditions. The questionnaire provided details on smoking status and hypertension treatment. To ascertain specific diagnoses and medications, a review of medical records was conducted. SLE disease activity and damage were measured using the Systemic Lupus Erythematosus Disease Activity Index-2000 (SLEDAI-2K) [10] and the Systemic Lupus International Collaborating Clinics/American College of Rheumatology (SLICC/ACR Damage Index-SDI-) [11], respectively. In this proposed study, the SLEDAI-2k index was categorized into different levels: none (0 points), mild (1–5 points), moderate (6–10 points), high (11–19 points), and very high activity (>20 points), as previously outlined [12]. Blood cell counts were measured using the Sysmex-XN automated blood cell analyzer (Sysmex, Kobe, Japan). Hematological profiles and disease scores were obtained during the same clinic visit. Dyslipidemia was defined by the presence of one or more of the following criteria: total cholesterol >200 mg/dL, triglycerides >150 mg/dL, HDL cholesterol <40 in men or <50 mg/dL in women, or LDL cholesterol >130 mg/dL. Additionally, patients with SLE, and not controls, underwent a carotid ultrasound to assess carotid intima–media wall thickness (cIMT) in the common carotid artery and to identify focal plaques in the extracranial carotid, following the Mannheim consensus definitions [13].

### 2.3. Statistical Analysis

Demographic and clinical characteristics of individuals with SLE were presented as mean (standard deviation) or percentages for categorical variables. For continuous variables that deviated from a normal distribution, data were expressed as median and interquartile range (IQR). Univariable differences between patients and controls were evaluated using Student’s *t*-test, the Mann–Whitney U-test, Chi-squared test, or Fisher’s exact test, depending on the normal distribution or the number of subjects. Differences between patients and controls in terms of hematological cell counts were analyzed through multivariable linear regression analyses, with controls serving as the reference variable. The association between disease-related data and MPV was explored using multivariable linear regression analysis, adjusting for confounding variables. Confounders were chosen from demographics and traditional cardiovascular risk factors if their *p*-values were below 0.20 in the univariable analysis. Additionally, we use the criterion of 10% of modification in beta coefficients when performing the multivariable analysis. All analyses were performed using Stata software, version 17/SE (StataCorp, College Station, TX, USA), with the two-sided significance level set at 5%. A *p*-value less than 0.05 was considered statistically significant.

## 3. Results

### 3.1. Demographic and Disease-Related Data of Patients with Systemic Lupus Erythematosus

Table 1 provides a summary of the characteristics of the 179 SLE patients and 181 age- and sex-matched controls included in the study. Most participants were women, comprising more than 90% of both study populations, with a mean age of 50 ± 16 and 50 ± 11 years in controls and SLE patients, respectively. The average body mass index was slightly lower in SLE patients compared to the control group, and this difference was statistically significant. Classic cardiovascular risk factors were prevalent in both the patient and control groups. Diabetes and dyslipidemia were significantly more common among controls. Additionally, there were no significant differences in statin use between the two groups, but aspirin use was higher in SLE patients. The cIMT in patients with SLE was 630 ± 110 microns, and carotid plaques were present in 34% of them (refer to Table 1).

The median duration of SLE was 17 ± 10 years. A majority of SLE patients exhibited either no disease activity (36%) or mild activity (34%), as evidenced by their SLEDAI-2K scores. The SLICC-SDI index revealed a median of 1 (IQR 0–2), with a noteworthy 70% of patients having a SLICC-SDI score of 1 or higher. Approximately 55% of patients were prescribed prednisone, with a median daily dose of 5 mg/day (IQR 5–7.5). At the time of recruitment, 60% of patients tested positive for anti-DNA antibodies, and 73% were positive for anti-ENA antibodies, with anti-SSA being the most commonly detected autoantibody (34%). Sixty-five percent of patients were using hydroxychloroquine during the study. Other disease-modifying antirheumatic drugs included methotrexate (10%) and azathioprine (21%). Further details regarding SLE-related information are presented in Table 1.

### 3.2. Multivariable Analysis of the Differences between Patients and Controls in Mean Platelet Volume

Table 2 displays complete blood cell count values for patients with SLE and controls. Numerous differences were observed after conducting multivariable analysis. Specifically, after adjustment, the total red blood cell count, hemoglobin, and hematocrit were significantly lower in SLE patients compared to controls. Regarding white blood cells, including leukocytes, neutrophils, lymphocytes, basophils, and eosinophils, all were significantly lower in patients with SLE compared to controls. However, there were no significant differences in monocyte levels between the two groups. Similarly, the number of platelets was significantly lower in SLE patients compared to controls. Remarkably, MPV was also significantly lower in patients with SLE compared to controls after multivariable analysis (beta coefficient, −0.7 [95% confidence interval −1.1 to −0.3)] fL, (*p* < 0.001)) (Table 2).

### 3.3. Demographic and Disease Characteristics in Relation to MPV

Demographic characteristics and cardiovascular risk factors were not significantly associated with MPV values. Dyslipidemia demonstrated a tendency to be associated with higher MPV values, although statistical significance was not achieved. Concerning disease characteristics, the damage inflicted by the disease, as measured by the SLICC index, exhibited a negative and significant relationship after multivariable adjustment with MPV (beta coef. −0.2 [95%CI −0.3 to −0.05) fL, *p* = 0.005). Furthermore, when the difference between the adjusted and unadjusted beta coefficients was analyzed, it was found to vary by less than 10%. This finding indicates that multivariable adjustment is not necessary in this case. Conversely, no correlations were observed between MPV and the SLEDAI score, autoimmunity profile or the treatment utilized for the disease (Table 3). Likewise, we did not observe a relationship between the presence of subclinical carotid atherosclerosis and MPV values.

Considering that SLICC and SLEDAI are composed by aggregating various aspects of the disease, we have examined the relationship between individual items within these scores and MPV. In the case of SLEDAI, the presence of hematuria and leukopenia emerged as the items significantly associated with lower MPV values (Table 4). Regarding SLICC, MPV values were significantly associated with several items from different domains. Hence, the presence of proteinuria and decreased glomerular filtration rate was associated with significantly lower MPV values. Similarly, the domain of peripheral vascular disease also showed a significant relationship with lower MPV values. This was also the case for the presence of arthritis or having 1 or more than 1 point in the musculoskeletal domain, for which lower MPV values were also found (Table 5).

## 4. Discussion

This work represents the most complete study in which MPV has been evaluated in patients with SLE. According to our results, MPV is lower in patients with SLE compared to controls. Furthermore, MPV is related, after multivariate analysis, to the cumulative damage caused by the disease. Specifically, renal, vascular, and musculoskeletal manifestations may be the disease characteristics associated with decreased MPV in patients with SLE.

Previous studies on MPV in SLE have been scarce and with contradictory results. In a study of 39 female patients with SLE and 45 controls, MPV was significantly higher in patients compared to controls [14]. Furthermore, in this report, MPV was significantly higher in patients with arthritis, nephritis, central nervous system involvement, vasculitis, and SLEDAI [14]. However, in a meta-analysis of nine studies that included 376 active SLE patients and 270 inactive SLE patients, no significant difference in MPV level was found between both groups [15]. Some studies have assessed the relation of MPV with disease activity. For instance, in a study with 128 adults with SLE who were assigned into two subgroups (99 with active phase and 29 with inactive phase), the mean MPV level in active patients was significantly higher than that in inactive subjects [16]. On the other hand, in a study of 36 patients with SLE with kidney involvement and proteinuria in the nephrotic range, who were studied in the active and remission periods of the disease between 2005 and 2013, the mean MPV in the active disease phase was statistically significantly higher than in the remission period [17]. Contrary, in a report that compared two groups of adults stratified according to disease activity (36 per group), MPV was significantly decreased in those SLE patients with active disease [18]. Moreover, a recent meta-analysis did not support the use of MPV as an indicator for monitoring disease activity in SLE patients [15]. Our results coincide with this meta-analysis as we did not observe a relationship between the SLEDAI activity index and MPV. Furthermore, in our study and unlike in previous ones, we performed a multivariate analysis to avoid possible confounding factors. Likewise, the number of subjects recruited in our study was considerably larger than that of the aforementioned studies. In addition, we performed a comprehensive characterization of the patients, considering the individual items of each score and multiple aspects of the disease, such as the autoimmune profile, comorbidity, and the treatments used.

To the best of our knowledge, our study constitutes the first attempt to evaluate the relationship between cumulative disease damage, through the SLICC score, and MPV. In our work, we found a negative and significant relationship, after adjustment, between the MPV and this score. Moreover, when the disease items of this index were analyzed separately, we observed that this relationship was fundamentally driven by the presence of renal, vascular, and musculoskeletal manifestations. This was partially described before. In this regard, MPV was reported to be significantly lower in 44 SLE patients with arthritis and active disease compared to another 44 SLE patients who had had arthritis but were in a period of inactive disease [19]. Taken together, we think that all the manifestations found to be associated with MPV in our study may have an influence on MPV in patients with SLE.

Some previous studies have described that MPV values are higher in patients with retinal vein occlusion [20] or diabetic retinopathy [21], suggesting that larger platelets may contribute to the pathogenesis of these disorders. However, we found a positive, and not negative, relationship between the item retinal change or optic atrophy and MPV values. However, it should be noted that only 9 subjects in our series presented this retinal pathology. Therefore, additional studies, including larger series of SLE patients with retinal involvement, are needed to elucidate the value of MPV in identifying the risk of retinal damage in SLE.

We do not have an exact answer to justify the biological basis behind a lower MPV in patients with SLE and its relation to accrual disease damage. Perhaps, the presence of inflammation due to disease damage leads to an increased demand for platelets, and the bone marrow may release younger and larger platelets, resulting in an increase in MPV values. Additionally, some disorders associated with disease damage can affect platelet production, activation, and destruction, which, in turn, can influence MPV values. Furthermore, SLEDAI activity, which is related to short-term activity, may not have sufficient influence to change MPV values.

We acknowledge several limitations in our study. In this sense, it was cross-sectional and, therefore, causality could not be inferred. Furthermore, therapies used for the treatment of SLE often influence hematological findings. However, in our study, we assessed the different treatments used by SLE patients and we were not able to establish a relationship between these therapies and MPV. Moreover, we recognize that we did not collect information regarding erythrocyte sedimentation rate in our study. Erythrocyte sedimentation rate has been described as a more accurate biomarker in patients with SLE compared to CRP. For this reason, we cannot conclude how erythrocyte sedimentation rate relates to MPV in patients with SLE. However, erythrocyte sedimentation rate and CRP are generally correlated with each other. For this reason, we believe that ESR may not show correlation with MPV in SLE. Nevertheless, this relationship will have to be studied in future studies. Also, most of the patients were in the no and low disease activity category. Maybe this was the reason for not finding a relation between the SLEDAI score and MPV. The finding regarding a lack of association between SLEDAI and MPV would need to be replicated in the future. Carotid assessment was not performed in our work. For this reason, we cannot conclude how MPV related to carotid values in controls. Dyslipidemia and diabetes were more frequent in controls compared to SLE patients. We do not have an exact explanation for this. However, this has not affected our results since these variables were included in the multivariable analysis. Finally, our results would need to be replicated in other rheumatological diseases to confirm if MPV also has this role in other autoimmune disorders.

In conclusion, MPV values are lower in patients with SLE compared to controls. Renal, vascular, and musculoskeletal manifestations correlate with reduced MPV levels. MPV could potentially function as a biomarker to assess disease damage in SLE.

## Figures and Tables

**Table 1:** Characteristics of systemic lupus erythematosus patients and controls.

	Controls	SLE Patients	
	(*n* = 181)	(*n* = 179)	*p*
Age, years	49.8 ± 16.1	49.9 ± 11.6	0.89
Female, *n* (%)	162 (90)	163 (91)	0.62
Body mass index, kg/m^2^	**30 ± 3**	**28 ± 6**	**<0.001**
Cardiovascular co-morbidity			
Diabetes, *n* (%)	**28 (16)**	**13 (7)**	**0.012**
Obesity, *n* (%)	49 (27)	64 (36)	0.076
Smoking, *n* (%)	32 (17)	41 (23)	0.22
Hypertension, *n* (%)	51 (28)	66 (37)	0.072
Dyslipidemia, *n* (%)	**140 (77)**	**118 (66)**	**0.016**
Carotid plaque, *n* (%)		57 (34)	
Carotid intima media thickness, microns		630 ± 110	
SLE related data			
Disease duration, years		17 ± 10	
CRP, mg/dL		2.0 (1.0–4.5)	
SLICC-DI		1 (0–2)	
SLICC-DI ≥1, *n* (%)		126 (70)	
SLEDAI-2K		2 (0–6)	
SLEDAI categories, *n* (%)			
No activity		64 (36)	
Mild		60 (34)	
Moderate		38 (22)	
High		10 (6)	
Very High		4 (2)	
Auto-antibody profile, *n* (%)			
Anti-ENA positive		108 (73)	
Anti-histone		22 (15)	
Anti-nucleosome		32 (22)	
Anti-ribosome		13 (9)	
Anti-SSA		50 (34)	
Anti-SSB		4 (3)	
Anti-Sm		7 (5)	
Anti-RNP		36 (24)	
Anti-DNA positive		74 (60)	
Antiphospholipid syndrome, *n* (%)		36 (20)	
Antiphospholipid autoantibodies, *n* (%)		43 (36)	
ACA IgG, *n* (%)		19 (16)	
ACA IgM, *n* (%)		9 (8)	
Lupus anticoagulant, *n* (%)		24 (26)	
Anti beta2 glycoprotein IgM, *n* (%)		8 (7)	
Anti beta2 glycoprotein IgG, *n* (%)		20 (18)	
Statins, *n* (%)	44 (24)	47 (26)	0.67
Aspirin, *n* (%)	**9 (11)**	**53 (29)**	**0.001**
Current prednisone, *n* (%)		98 (55)	
Prednisone, mg/day		5 (5–7.5)	
Methotrexate, *n* (%)		18 (10)	
Mycophenolate mofetil, *n* (%)		24 (13)	
Hydroxychloroquine, *n* (%)		117 (65)	
Azathioprine, *n* (%)		37 (21)	
Rituximab, *n* (%)		4 (2)	
Belimumab, *n* (%)		7 (4)	

The data are presented as mean ± SD or median (interquartile range) in cases where the distribution was not normal. BMI stands for body mass index, and additional abbreviations include C3 C4 for complement, CRP for C-reactive protein, LDL for low-density lipoprotein, ACA for anticardiolipin, ANA for antinuclear antibodies, and ENA for extractible nuclear antibodies. SLEDAI refers to the Systemic Lupus Erythematosus Disease Activity Index. SLICC represents the Systemic Lupus International Collaborating Clinics/American College of Rheumatology Damage Index. Statistically significant *p*-values are indicated in bold.

**Table 2:** Multivariable analysis of the differences between patients and controls in mean platelet volume.

	Controls	SLE Patients			
	(*n* = 181)	(*n* = 179)	*p*	Beta Coef. (95%CI)	*p*
	Univariable			Multivariable	
Leucocytes/mm^3^	**7356 ± 1972**	**6117 ± 2416**	**<0.001**	**−1070 (−1712–(−429))**	**0.001**
Neutrophils/mm^3^	**4019 ± 1496**	**3648 ± 1872**	**0.039**	−373 (−871–126)	0.14
Lymphocytes/mm^3^	**2479 ± 875**	**1742 ± 1068**	**<0.001**	**−567 (−838–(−295))**	**<0.001**
Monocytes/mm^3^	582 ± 169	556 ± 2113	0.20	−19 (−74–37)	0.51
Eosinophils/mm^3^	**191 (110–293)**	**110 (70–180)**	**<0.001**	**−86 (−126–(−46))**	**<0.001**
Basophils/mm^3^	**43 (32–61)**	**30 (20–40)**	**<0.001**	**−15 (−24–(−6))**	**0.001**
Red blood cells, ×10 × 10^6^/mm^3^	**4.69 ± 0.43**	**4.48 ± 0.46**	**<0.001**	**−0.2 (−0.3–(−0.06))**	**0.003**
Hemoglobin, g/dL	**13.7 ± 1.3**	**13.2 ± 1.4**	**<0.001**	**−0.5 (−0.9–(−0.06))**	**0.023**
Hematocrit, %	**42.1 ± 3.4**	**40.2 ± 3.9**	**<0.001**	**−1.6 (−2.7–(−0.58))**	**0.003**
Mean corpuscular volume, fL	89.8 ± 5.5	89.8 ± 6.0	0.93	0.1 (−1.5–1.7)	0.89
Mean corpuscular hemoglobin, pg	29.3 ± 2.2	29.5 ± 2.5	0.43	0.3 (−0.4–1)	0.36
Mean corpuscular hemoglobin concentration, g/dL	**32.5 ± 1.1**	**32.8 ± 1.2**	**0.034**	0.3 (−0.06–0.6)	0.10
Red Cell Distribution Width, %	**13.4 ± 1.5**	**14.4 ± 2.2**	**<0.001**	**1.3 (0.8–1.8)**	**<0.001**
Platelets ×10 × 10^3^/mm^3^	**270 ± 60**	**240 ± 77**	**<0.001**	**−28 (−48–(−86))**	**0.005**
Mean platelet volume, fL	**10.1 ± 1.2**	**9.6 ± 1.6**	**<0.001**	**−0.7 (−1.1–(−0.3))**	**<0.001**

In the multivariable analysis, controls are considered the reference variable. Significant *p* values are depicted in bold. Multivariable analysis is adjusted for body mass index, diabetes, hypertension, dyslipidemia, and aspirin intake.

**Table 3:** Relationship of demographic and disease characteristics to mean platelet volume.

	Mean Platelet Volume, fL			
	Univariable		Adjusted	
	Beta Coef. (95%), *p*		Beta Coef. (95%), *p*	
Age, years	0.005 (−0.02–0.03)	0.62		
Female	−0.07 (−1–0.1)	0.11		
Body mass index, kg/m^2^	0.03 (−0.008–0.07)	0.12		
Cardiovascular co-morbidity				
Smoking	0.4 (−0.2–0.9)	0.17		
Diabetes	−0.05 (−0.9–0.8)	0.92		
Hypertension	−0.2 (−0.7–0.3)	0.44		
Obesity	0.2 (−0.3–0.7)	0.46		
Dyslipidemia	0.4 (−0.06–0.9)	0.085		
Statins	−0.3 (−0.8–0.2)	0.26		
Aspirin	−0.03 (−0.5–0.5)	0.91		
Carotid intima media thickness, mm	0.6 (−1–3)	0.55		
Carotid plaque	0.1 (−0.4–0.6)	0.65		
SLE related data				
Disease duration, years	0.009 (−0.01–0.03)	0.46		
CRP, mg/dL	−0.008 (−0.03–0.009)	0.34		
SLICC-DI	**−0.2 (−0.3–(−0.06))**	**0.003**	**−0.2 (−0.3–(−0.05))**	**0.005**
SLICC-DI ≥1	−0.4 (−0.9–0.1)	0.11	−0.4 (−0.9–0.09)	0.11
SLEDAI	−0.01 (−0.06–0.04)	0.62		
SLEDAI categories, *n* (%)				
No activity	ref.			
Mild	0.1 (−0.4–0.7)	0.68		
Moderate to very high	−0.04 (−0.6–0.5)	0.89		
Auto-antibody profile				
Anti-DNA positive	−0.04 (−0.6–0.5)	0.88		
Anti-ENA positive	−0.4 (−1–0.2)	0.18	−0.3 (−0.9–0.2)	0.24
Anti-SSA	−0.2 (−0.7–0.4)	0.57		
Anti-SSB	0.5 (−1–2)	0.52		
Anti-RNP	0.04 (−0.6–0.6)	0.89		
Anti-Sm	−0.3 (−2–0.9)	0.61		
Anti-ribosome	−0.08 (−1–0.8)	0.87		
Anti-nucleosome	−0.1 (−0.8–0.5)	0.67		
Anti-histone	−0.2 (−0.9–0.5)	0.56		
Antiphospholipid syndrome	0.4 (−0.2–0.9)	0.21		
Antiphospholipid autoantibodies	0.3 (−0.3–0.9)	0.32		
Lupus anticoagulant	0.3 (−0.5–1)	0.44		
ACA IgM	0.6 (−0.6–2)	0.33		
ACA IgG	0.2 (−0.6–1)	0.55		
Anti beta2 glycoprotein IgM	0.002 (−1–1)	0.99		
Anti beta2 glycoprotein IgG	0.4 (−0.3–1)	0.27		
Current prednisone	−0.2 (−0.6–0.3)	0.45		
Prednisone, mg/day	−0.06 (−0.1–0.03)	0.20		
Hydroxychloroquine	0.04 (−0.4–0.5)	0.88		
Methotrexate	−0.4 (−1–0.4)	0.30		
Mycophenolate mofetil	−0.1 (−0.8–0.5)	0.69		
Azathioprine	−0.3 (−0.9–0.3)	0.28		
Rituximab	−0.007 (−2–2)	0.99		
Belimumab	0.9 (−0.3–2)	0.14	0.8 (−0.4–2)	0.20

In this analysis, MPV serves as the dependent variable. The multivariable analysis is adjusted for sex, body mass index (BMI), smoking, and dyslipidemia. Additional abbreviations include CRP for C-reactive protein, ACA for anticardiolipin, ANA for antinuclear antibodies, and ENA for extractible nuclear antibodies. SLEDAI refers to the Systemic Lupus Erythematosus Disease Activity Index. SLICC stands for the Systemic Lupus International Collaborating Clinics/American College of Rheumatology Damage Index. Statistically significant *p*-values are highlighted in bold.

**Table 4:** Relationship of SLEDAI elements to MPV values.

			MPV, fL	
	*n*	%	Beta Coef. (95%), *p*	
Seizures	1	1	0.4 (−3–4)	0.79
Psychosis	1	1	1 (−2–5)	0.37
Organic brain syndrome	0	0	-	
Visual disturbance	1	1	**3 (0.2–6)**	**0.038**
Cranial nerve disorder	1	1	−2 (−5–1)	0.31
Lupus headache	1	0	1 (−2–5)	0.37
ACVA	0	0	-	
Vasculitis	1	1	−3 (−6–0.5)	0.097
Arthritis	9	5	−0.5 (−2–0.6)	0.35
Myositis	0	0	-	
Urinary cylinders	7	4	−1 (−2–0.1)	0.074
Hematuria	14	8	**−2 (−2–(−0.8))**	**<0.001**
Proteinuria	5	3	−1 (−3–0.08)	0.066
Pyuria	10	6	−0.5 (−1–0.5)	0.37
Rash	16	9	−0.7 (−2–0.09)	0.082
Alopecia	8	4	0.2 (−0.9–1)	0.69
Mucosal ulcers	10	6	−0.2 (−1–0.8)	0.75
Pleurisy	1	1	−3 (−6–0.5)	0.097
Pericarditis	0	0	-	
Low complement	49	28	0.4 (−0.1–0.9)	0.12
Elevated anti-DNA	62	35	0.2 (−0.3–0.7)	0.38
Fever	2	1	−1 (−3–1)	0.32
Thrombopenia	7	4	1 (−0.1–2)	0.083
Leukopenia	17	10	**−1 (−2–(−0.3))**	**0.009**

Mean platelet volume (MPV) is the dependent variable in this analysis. ACVA: Acute cerebrovascular accident. Significant *p* values are depicted in bold.

**Table 5:** Relationship of SLICC scoring elements with MPV.

			MPV, fL	
	*n*	%	Beta Coef. (95%), *p*	
Ocular				
Any cataract ever	22	13	0.2 (−0.5–0.9)	0.52
Retinal change or optic atrophy	15	9	**0.9 (0.1–2)**	**0.025**
Points ≥ 1 in the domain	35	20	0.2 (−0.4–0.8)	0.55
Neuropsychiatric				
Cognitive impairment	3	2	−0.6 (−2–1)	0.51
Transverse myelitis	1	1	2 (−0.6–5)	0.12
Cranial or peripheral neuropathy	3	2	−0.6 (−2–1)	0.51
Cerebrovascular accident ever	11	6	−0.2 (−1–0.7)	0.47
Seizures requiring therapy for 6 months	10	6	−0.3 (−1–0.7)	0.50
Points ≥1 in the domain	24	13	−0.06 (−0.7–0.6)	0.86
Renal				
Proteinuria 3.5 gm/24 h	6	3	**−1 (−3–(−0.09))**	**0.036**
End-stage renal disease	2	1	−0.7 (−1–0.008)	0.053
Estimated or measured glomerular filtration rate <50%	9	5	**−1 (−2–(−0.2))**	**0.018**
Points ≥1 in the domain	18	10	**−1 (−2–(−0.6))**	**0.001**
Pulmonary				
Pulmonary hypertension	3	2	−1 (−3–0.5)	0.15
Pleural fibrosis	0	0	-	
Pulmonary infarction	0	0	-	
Pulmonary fibrosis	3	2	0.07 (−2–2)	0.94
Shrinking lung	0	0	-	
Points ≥1 in the domain	9	5	−0.8 (−2–0.2)	0.12
Cardiovascular				
Myocardial infarction ever	2	1	1 (−0.8–4)	0.20
Angina or coronary artery bypass	4	2	0.4 (−1–2)	0.60
Valvular disease	5	3	0.2 (−1–2)	0.77
Cardiomyopathy	1	1	−3 (−6–0.5)	0.095
Pericarditis for 6 months, or pericardiectomy	1	1	1 (−2–5)	0.37
Points ≥1 in the domain	13	7	−0.1 (−1–0.8)	0.80
Peripheral vascular				
Minor tissue loss (pulp space)	3	2	**−2 (−4–(−0.2))**	**0.027**
Significant tissue loss ever	0	0	-	
Venous thrombosis	11	7	−0.4 (−1–0.6)	0.41
Claudication for 6 months	3	2	−1 (−3–0.8)	0.28
Points ≥1 in the domain	25	14	**−0.7 (−1–(−0.07))**	**0.030**
Gastrointestinal				
Infarction or resection of bowel	15	8	−0.7 (−2–0.1)	0.11
Mesenteric insufficiency	1	1	−2 (−5–1)	0.31
Infarction or resection of bowel below duodenum, spleen, liver, or chronic peritonitis	0	0	-	
Stricture or upper gastrointestinal tract surgery ever	0	0	-	
Pancreatic insufficiency	0	0	-	
Points ≥1 in the domain	17	10	−0.7 (−2–0.04))	0.064
Musculoskeletal				
Muscle atrophy or weakness	3	2	−0.3 (−2–1)	0.73
Osteomyelitis	1	1	**3 (0.2–6)**	**0.039**
Deforming or erosive arthritis	33	20	**−1 (−2–(−0.4))**	**0.001**
Osteoporosis with fracture or vertebral collapse	15	9	−0.6 (−1–0.3)	0.18
Avascular necrosis	2	1	1 (−0.8–4)	0.21
Tendon rupture	3	2	−1 (−30.5)	0.15
Points ≥1 in the domain	64	36	**−0.7 (−1–(−0.3))**	**0.002**
Skin				
Scarring chronic alopecia	10	6	0.06 (−0.9–1)	0.91
Extensive scarring or panniculum	9	5	−0.5 (−2–0.6)	0.36
Skin ulceration	4	2	0.4 (−1–2)	0.59
Points ≥1 in the domain	29	16	−0.09 (−0.7–0.5)	0.77
Premature gonadal failure	9	5	0.02 (−1–1)	0.97
Diabetes (regardless of treatment)	13	7	−0.05 (−0.9–0.8)	0.92
Malignancy (exclude dysplasia)	6	3	−0.4 (−2–0.9)	0.58

Mean platelet volume (MPV) is the dependent variable in this analysis; SLICC items and domains represent the independent variable. Significant *p* values are depicted in bold. SLICC: Systemic Lupus International Collaborating Clinics/American College of Rheumatology Damage Index.

## Data Availability

The data sets used and/or analyzed in the present study are available from the corresponding author upon request.
